# Biological evaluation of transdichloridoplatinum(II) complexes with 3- and 4-acetylpyridine in comparison to cisplatin

**DOI:** 10.2478/raon-2013-0050

**Published:** 2013-10-08

**Authors:** Lana Filipovic, Sandra Arandelovic, Nevenka Gligorijevic, Ana Krivokuca, Radmila Jankovic, Tatjana Srdic-Rajic, Gordana Rakic, Zivoslav Tesic, Sinisa Radulovic

**Affiliations:** 1 Institute for Oncology and Radiology of Serbia, Belgrade, Serbia; 2 Faculty of Chemistry, University of Belgrade, Belgrade, Serbia

**Keywords:** angiogenesis, apoptosis, MMPs, MRC-5, *trans*-platinum(II)

## Abstract

**Background:**

In our previous study we reported the synthesis and cytotoxicity of two *trans*-platinum(II) complexes: *trans*-[PtCl_2_(3-acetylpyridine)_2_] (**1**) and *trans*-[PtCl_2_(4-acetylpyridine)_2_] (**2**), revealing significant cytotoxic potential of **2.** In order to evaluate the mechanism underlying biological activity of both *trans*-Pt(II) isomers, comparative studies versus cisplatin were performed in HeLa, MRC-5 and MS1 cells.

**Materials and methods.:**

The cytotoxic activity of the investigated complexes was determined using SRB assay. The colagenolytic activity was determined using gelatin zymography, while the effect of platinum complexes on matrix metalloproteinases 2 and 9 mRNA expression was evaluated by quantitative real-time PCR. Apoptotic potential and cell cycle alterations were determined by FACS analyses. Western blot analysis was used to evaluate the effect on expression of DNA-repair enzyme ERCC1, and quantitative real-time PCR was used for the ERCC1 mRNA expression analysis. *In vitro* antiangiogenic potential was determined by tube formation assay. Platinum content in intracellular DNA and proteins was determined by inductively coupled plasma-optical emission spectrometry.

**Results:**

Compound **2** displayed an apparent cytoselective profile, and flow cytometry analysis in HeLa cells indicated that **2** exerted antiproliferative effect through apoptosis induction, while **1** induced both apoptosis and necrosis. Action of **1** and **2,** as analyzed by quantitative real-time PCR and Western blot, was associated with down-regulation of ERCC1. Both *trans*-complexes inhibited MMP-9 mRNA expression in HeLa, while **2** significantly abrogated *in vitro* tubulogenesis in MS1 cells.

**Conclusions:**

The ability of **2** to induce multiple and selective *in vitro* cytotoxic effects encourages further investigations of *trans*-platinum(II) complexes with substituted pyridines.

## Introduction

Cisplatin (CDDP) represents the basis of combination chemotherapy regimens in solid tumors, although main drawbacks to its successful application are development of resistance and toxic side effects.^1^,^2^ Search for CDDP analogues with improved pharmacological properties by manipulation of the structure of ligands, has achieved a reduction in toxicity, but obtained limited success in broadening spectrum of activity.^3^–^5^ However, novel classes of platinum complexes including *trans*-Pt(II) compounds with planar amine ligands are able to exert cytotoxicity, equivalent or better to that of CDDP, and posses different mechanisms of antitumor action.^6^–^10^ Cytotoxicity data of 107 platinum compounds from the NCI human tumor panel recognized *trans*-platinum complexes, of structural formula [PtCl_2_(L)(L’)] with planar amine ligands (L and L’ may be the same or different), as unique group of *trans*-platinum drugs that had cytotoxicities similar to that of their *cis*-isomers and CDDP, and possessed a novel cytotoxicity profile.^11^ Search for new platinum compounds with the complementary or wider range of activity than CDDP, whose actions would be more selective toward cancer comparing to normal cells, and which would possess different targets than the traditional CDDP, is always attractive topic.^7^ Formation and persistence of DNA-adducts are considered vital in platinum drug induced cytotoxicity, and type of DNA-damage is determined by the nature of platinum-coordinating ligands.^4^,^12^,^13^ However, cellular sensitivity to platinum complexes is multifactorial, with some of major mechanisms being the proficiency of the cellular mechanism for adducts recognition and repair, and the ability of cells to reduce intracellular platinum, due to deactivation by sulphur-containing biomolecules and/or drug efflux.^14^–^16^ Numerous studies imply the significance of copper transporters in regulating cellular pharmacology of CDDP by mediating its uptake and efflux in different cell lines.^17^

Structure-activity studies up to date demonstrated that bulky amine carrier ligands, such as pyridine, appear to sterically hinder approach of incoming nucleophiles to the axial positions of the platinum center, thus reducing deactivation of platinum by sulphur-containing biomolecules.^18^*Trans*-orientation of planar pyridines seems to contribute to greater affinity of complexes for interstrand cross-link formation and DNA conformational distortion, comparing to *cis*-isomers, leading to activation of different DNA-repair mechanisms.^19^–^21^

Recent studies demonstrated that fine tuning of the biological activity of *trans*-platinum pyridines may be achieved by different positioning of the substituents such as: methyl-group and 3- or 4-hydroxymethyl-group, on the pyridine ring.^19^,^20^,^22^

**FIGURE 1.**
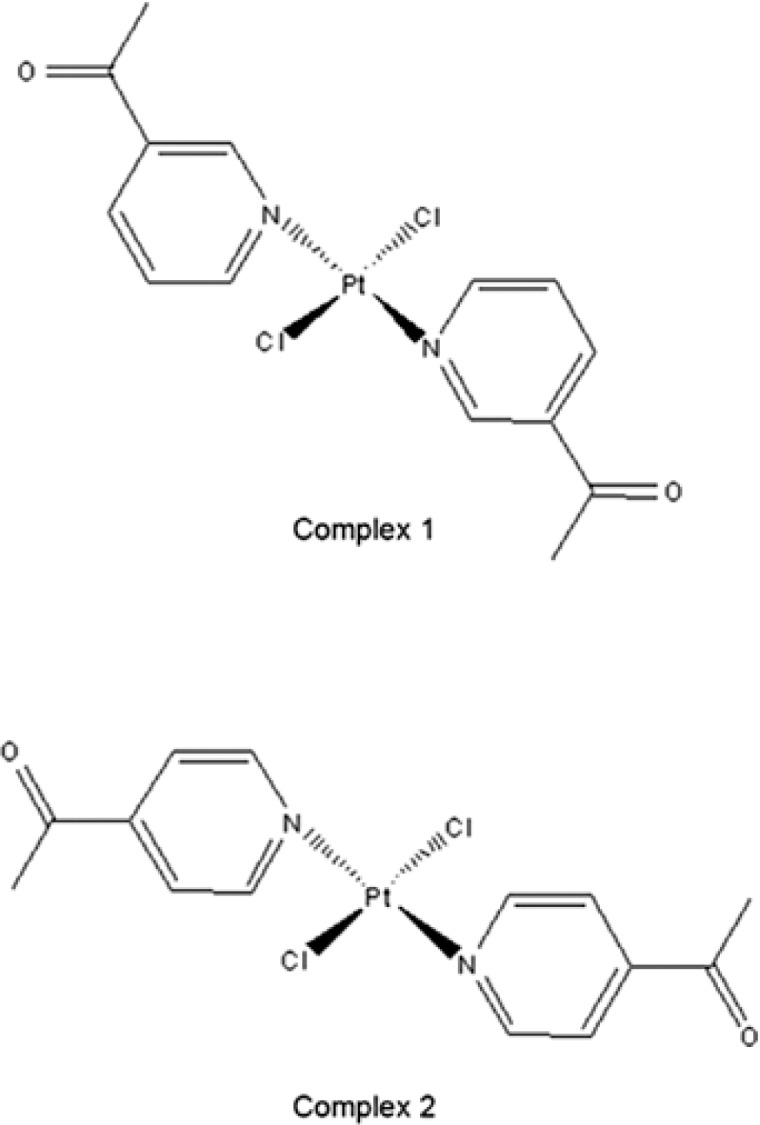
Structures of the investigated *trans*-platinum(II) complexes: *trans*-[PtCl_2_(3-acetylpyridine)_2_] **1**; *trans*-[PtCl_2_(4-acetylpyridine)_2_] **2**.

In our previous investigations, we have synthesized and characterized two *trans*-platinum(II) complexes, of structural formula *trans*-[PtCl_2_(L)_2_] with substituted pyridine ligands, L=n-acetylpyridine, (n = 3 or 4) (Figure 1). Cytotoxicity evaluation on the panel of tumor cell lines revealed potential of *trans-*[PtCl_2_(4-acetylpyridine)_2_] to exert activity in low micromolar range, with the highest cytotoxicity in HeLa cells, comparable to that of CDDP.^23^ Aim of this study was to investigate the molecular mechanisms underlying the *in vitro* biological activity of *trans*-[PtCl_2_(4-acetylpyridine)_2_] (complex **2**) and its less cytotoxic structural isomer *trans*-[PtCl_2_(3-acetylpyridine)_2_] (complex **1**), and to understand possible relations to their structural characteristics, such as the position of the acetyl substituent on the pyridine ring. Mechanistic studies were performed in comparison to CDDP in human cervix carcinoma cell line (HeLa), and two other cell lines: human normal lung fibroblast (MRC-5) cell line, which was used as a non-cancerous model system for *in vitro* toxicity evaluation, and murine endothelial cells immortalized by infection with a retrovirus encoding SV40 large T antigen (MS1), as a model system for *in vitro* testing of antiangiogenic effect.24 In order to test if the cytotoxic responses produced by compounds **1** and **2** in HeLa cells correlated with the platinum content in cellular DNA and to evaluate the mechanism of cytotoxic action, we studied the ability of complexes to bind intracellular DNA and proteins, and to induce DNA-damage related response, cell cycle alterations and apoptosis.

Based on the literature data reporting inhibitory effect of some platinum(II) compounds on matrix metalloproteinases (MMP) activity, we assumed that *trans*-[PtCl_2_(n-acetylpyridine)_2_] (n = 3 or 4), may possess ability to modulate diverse cellular processes, including those related to the cancer cell angiogenic and metastatic behaviour.^25^–^27^ Thus, in the final part of our study, we analyzed if the tested complexes affected gelatinolitic activity and mRNA expression of secreted forms of MMP-2 and MMP-9, or abrogated process of angiogenesis *in vitro.*

## Materials and methods

### Synthesis

Platinum complexes *trans*-[PtCl_2_(3-acetylpyridine)_2_] (complex **1**) and *trans*-[PtCl_2_(4-acetylpyridine)_2_] (complex **2**) (Figure 1) were synthesized and characterized by IR and NMR, as previously described.^23^

### Cytotoxic activity

#### Cell culture

HeLa and MRC-5 cells cells were maintained as monolayer culture in nutrient medium, Roswell Park Memorial Institute 1640 medium (RPMI1640), (Sigma-Aldrich Co).^28^ MS1 cells were maintained as monolayer culture in nutrient medium, Dulbecco`s Modified Eagle Medium (DMEM), (Sigma-Aldrich Co). Nutrient medium conditions and cell maintenance procedures were explained previously.^29^

### In vitro cytotoxicity assay (SRB)

Cells were seeded into 96-well plates (Thermo Scientific Nunc^™^), in number of 7000 cells per well (c/w) for MS1 and 5000 cells per well for MRC-5, and left for 24 h before complexes **1**, **2** and CDDP were added. Preparation of test solutions was performed immediately before experiments by dissolving in DMSO. The cells were treated with serial dilutions of the studied compounds for 48 h. Final concentrations achieved per wells were 1 μM, 3 μM, 10 μM, 30 μM and 100 μM. Each concentration was tested in triplicates, and the final concentration of DMSO solvent never exceeded 0.33%. Cytotoxicity of the investigated platinum complexes, and CDDP as a referent compound, was evaluated after 48 h of continuous action, using sulforhodamine B (Sigma–Aldrich Co.) colorimetric assay.^30^ The percentages of surviving cells relative to untreated controls were determined. The IC_50_ value, defined as the concentrations of the compound causing 50% cell growth inhibition, was estimated from the dose-response curves.

### Flow cytometric analysis of cell cycle phase distribution

Quantitative analysis of cell cycle phase distribution was performed by flow-cytometric analysis of the DNA content in fixed HeLa cells, after staining with propidium iodide (PI).^31^ Cells were seeded at density of 2×10^5^ into 6-well plates (Thermo Scientific Nunc^™^), and grown in nutrient medium. After 24 h cells were exposed to the investigated compounds **1**, **2** and CDDP for 24 h, at concentrations corresponding to IC_50_ or 1.5×IC_50_. The detailed procedure was previously described.^29^ Cell cycle phase distribution was analyzed using a fluorescence activated cell sorting (FACS) Calibur Becton Dickinson flow cytometer and Cell Quest computer software.

### Statistical analysis

Calculations of mean, SD, and p values were performed on triplicate experiments. The Student t-test was used to calculate p-values for comparison. The significant statistics was set at a p-value <0.05 (Stata Software).

### Annexin V-FITC apoptotic assay

Quantitative analysis of apoptotic and necrotic cell death induced by the investigated platinum complexes and CDDP, as a referent compound, was performed by Annexin V-FITC apoptosis detection kit, according to the manufacturer’s instructions (BD Biosciences). Precisely, 2×10^5^ HeLa cells treated with 1×IC_50_ of the tested compounds and CDDP for 4 and 24 h and the analysis was performed as previously reported.^29^

### Measurement of platinum binding to intracellular DNA or proteins using ICP-OES

Binding of platinum(II) to cellular DNA and proteins was analyzed in HeLa cells, using inductively coupled plasma optical emission spectrometry (ICP-OES). 6×10^6^ cells were seeded into 75 cm^2^ dish (Thermo Scientific Nunc^™^) and treated with the investigated complexes in concentrations corresponding to 0.5×IC_50_. Following 6 or 24 h, cells were harvested by scraping, washed by ice cold PBS and cell pellet was collected by centrifugation at 2000 rpm, 10 min. DNA and proteins were isolated using TRI Reagent® (Sigma-Aldrich Co.) according to the manufacturer’s procedure and concentrations were determined spectrophotometrically by measuring absorbance at A260 and A280 nm respectively (Eppendorf BioPhotometer 6131). Platinum(II) levels were determined in isolated DNA and protein fractions according to the standard procedure, using Thermo Scientific iCAP 6500 Duo ICP (Thermo Fisher Scientific).

### Quantitative real-time PCR (qRT-PCR)

#### Sample preparation for qRT-PCR; RNA extraction and cDNA synthesis

6×10^6^ HeLa cells were seeded in nutrient medium and after 24 h treated with the investigated complexes **1**, **2** or CDDP at concentrations corresponding to 0.5×IC_50_, for 6 and 24 hours. After treatment, cells were washed with ice cold PBS and harvested by scraping, while cell pellet was collected by centrifugation. Total RNA was isolated using TRI Reagent® (Sigma-Aldrich Co.) according to manufacturer’s recommendations. RNA extraction in details and cDNA synthesis were described earlier.^29^

#### Quantitative real-time PCR

The analysis of gene expression level of several genes and GAPDH (endogenous control) was done by using TaqMan® Gene Expression Assays for human genes (assay ID Applied Biosystem, listed as following: Hs_01012155_g1 (ERCC1); Hs_01548727_m1 (MMP-2); Hs_00234579_m (MMP-9); Hs_00355782_ m1 (GAPDH)) on ABI PRISM® 7500 PCR instrument (Applied Biosystems). PCR was performed as previously reported.^29^

### Western blot

HeLa cells were treated with the investigated platinum complexes **1** and **2** or CDDP for 6 h, at concentrations corresponding to the 0.5×IC_50_ values obtained for 48 h of continuous treatment. Cells maintained in nutrient medium were used as the untreated control. Sample preparation and the analysis were performed as described in the previous study.^29^,^32^ Purified mouse anti-human ERCC1 monoclonal antibody (1:500 dilution) (BD Biosciences Pharmingen) was used, as well as the secondary anti-mouse IgG-peroxidase conjugated antibody (1:2000 dilution) (Sigma-Aldrich Co.).

### Gelatin zymography

Effect of the investigated *trans-*Pt(II) complexes and CDDP on gelatinolitic activity of secreted matrix metalloproteinases MMP-2 and MMP-9 in HeLa was analyzed by zymography in 10% SDS-polyacrylamide gels impregnated with 0.1% gelatin.^33^ HeLa cells were treated with the complexes (0.5×IC_50_) for 6 h in serum-free medium and the precise conditions, as well as the procedure have been given previously.^29^ The gelatinolytic activities were visualized as clear transparent bands against the blue background of Coomassie brilliant blue-stained gelatin.

### Tube formation assay (*in vitro* angiogenesis assay)

Potential of *trans*-platinum(II) complexes and CDDP to inhibit angiogenesis *in vitro* was analyzed by tube formation assay in MS1 cells. MS1 cells, when plated into gel of basement membrane proteins, rapidly organize into multicellular tube-like structures, while antiangiogenic effect of the tested compounds is observed as the reduction of tube formation.^24^ Briefly, 24-well plates were coated with collagen and allowed to solidify at 37°C 1 h. MS1 cells were seeded into wells (1×10^5^ c/w) in nutrient medium. Complexes **1** and **2** were added 2 h after cells settled, at concentrations corresponding to 0.03×IC_50_, which was non-toxic to the cells. Tube formation was observed periodically over time under microscope and representative pictures were taken after 24 h incubation with Olympus digital camera connected to the inverted microscope (Carl Zeiss, Jena, Germany, objective 6.3/0.20).

### Morphological examination by light microscopy

HeLa cells (50000 c/w) and MRC-5 cells (125000 c/w), were seeded into 6-well plates (Thermo Scientific Nunc^™^), in the corresponding nutrient medium, and after 24 h of growth cells were exposed to complexes **1**, **2** or CDDP, at equimolar concentrations of 5 mM. Following 24 h of treatment, cells were observed under the light microscope and photographs were taken with Olympus digital camera connected to the inverted microscope (Carl Zeiss, Jena, Germany, objective 6.3/0.20)..

### Statistical analysis

Statistical comparison of IC_50_ values in MRC-5 cell line versus HeLa and MS1 cell line, was performed using one way statistical analysis of variance (one-way ANOVA - GraphPad Software). IC_50_ values were determined as mean ± SD (standard deviation) of three or more independent experiments.

## Results

### *In vitro* cytotoxicity assay (SRB)

**FIGURE 2.**
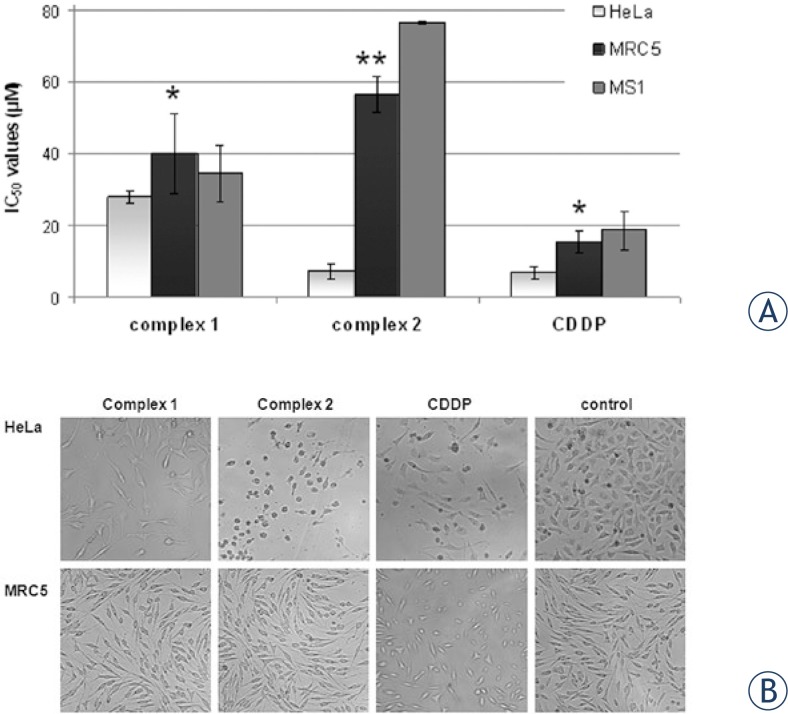
**A** Diagram presenting cytotoxicity of the tested agents and cisplatin in terms of IC_50_ values, obtained for 48 h of drug action, by SRB assay. IC_50_ values present average (±SD) obtained from three or more independent experiments. Asterisks denotes p values, when comparing MRC-5 cells to HeLa cells, by ANOVA test: **1** (*) p > 0.05; **2** (**) p < 0.001; CDDP (*) p > 0.05; **B** Micrographs of HeLa cells or MRC-5 cells exposed to equimolar (5 mM) concentration of tested platinum complexes **1**, **2** or CDDP, following 24 h treatment, versus control (non treated cells). Micrographs are one representative experiment selected of three and were obtained with Olympus digital camera connected to the inverted microscope (Carl Zeiss, Jena, Germany, objective 6.3/0.20).

In order to further investigate cytotoxic and cytoselective potential of the two *trans*-platinum isomers, in comparison to CDDP, growth inhibitory study was performed in MRC-5 cells, which were used as non-cancerous model for *in vitro* toxicity evaluation; and MS1 cells as *in vitro* model for testing of antiangiogenic effect. Cytotoxicity of the complexes summarized in terms of IC_50_ values, is presented in Figure 2A. IC_50_ values (mM) obtained for 48 h of continuous drug action in MRC-5 cells, may be arranged in increasing order as following: 15.4 ± 3.1 mM, for CDDP; 40.0 ± 11.1 mM for complex **1**; and 56.4 ± 5.0 mM for **2**, indicating lower toxicity of *trans*-complexes in non-cancerous cell model comparing to CDDP. Particularly, complex **2** exerted less cytotoxicity in MRC-5 cells than in HeLa, by a factor of approximately four-fold, indicating significant cytoselective potential toward neoplastic cells (p < 0.001). Both *trans*-complexes exhibited poor activity, in MS1 cells with IC_50_ values being: 76.3 ± 0.5 mM for complex **2**; 34.5 ± 7.8 mM for complex **1**; comparing to CDDP (IC_50_ 18.6 ± 5.4 mM).

### Morphological examination

The results of the morphological analysis of HeLa and MRC-5 cells are presented in Figure 2B, as micrographs obtained following 24 h agents action. Results indicated that in the presence of 5 mM of complex **2**, concentration which corresponded to IC_50_ value in HeLa cells, viability of MRC-5 cells was not significantly altered, suggesting cytoselective potential toward neoplastic HeLa cells. Oppositely, complex **1** haven’t exerted cytoselective potential, as observed in Figure 2B. Morphological changes of MRC-5 cells, such as: cell shrinkage and detachment, following equimolar treatment with CDDP, were indicative for apoptosis.

### Determination of cell cycle perturbation by flow cytometry

**FIGURE 3.**
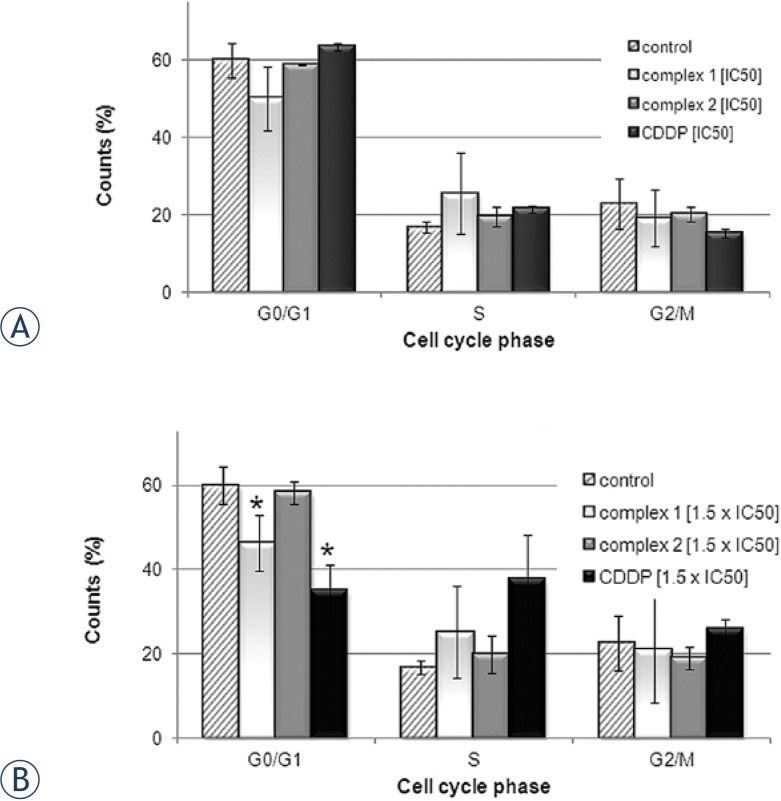
Diagrams presenting cell cycle phase distribution of treated HeLa cells, obtained by flow-cytometric analysis of the DNA content in fixed cells, after staining with PI. HeLa cells were collected following 24 h treatment with tested complexes or cisplatin at concentration corresponding to **A** IC_50_ and **B** 1.5×IC_50_. Bar graphs represent mean ± SD in at least three independent experiments. Asteriks (*) denotes p values < 0.05, calculated by Student t-test, indicating statisticaly significant differences (Stata Software).

The potential of the tested complexes to induce cell cycle alterations in comparison to CDDP in HeLa cells, was examined by flow cytometry using staining with PI. Results are presented as diagrams of cell distribution over the cell cycle phases after 24 h of agent action, where Figure 3A shows effects of the complexes at concentration corresponding to IC_50,_ and Figure 3B shows effects of the complexes at concentration corresponding to 1.5×IC_50_. Complex **1**, induced arrest in the S phase of cell cycle at concentrations corresponding to 1.5 × IC_50_, but less than CDDP (Figure 3B). Complex **1** induced decrease of cell percentage in the G0/G1 phase in concentration dependent manner, comparing to the non-treated control. CDDP induced dose-dependent arrest in the S phase of cell cycle, and decrease of cell progression through G2/M phase (Figures 3A and 3B).

The Student t-test showed that the difference is considered to be statisticaly significant only for G0/G1 cells when two groups of cell cycle results were compared (control cells compared to cells treated with 1.5×IC_50_. complex **1**, and control cells compared to cells treated with 1.5×IC_50_. CDDP (p<0.05, Stata Software)).

### Quantification of apoptosis by annexin V-FITC binding

**FIGURE 4.**
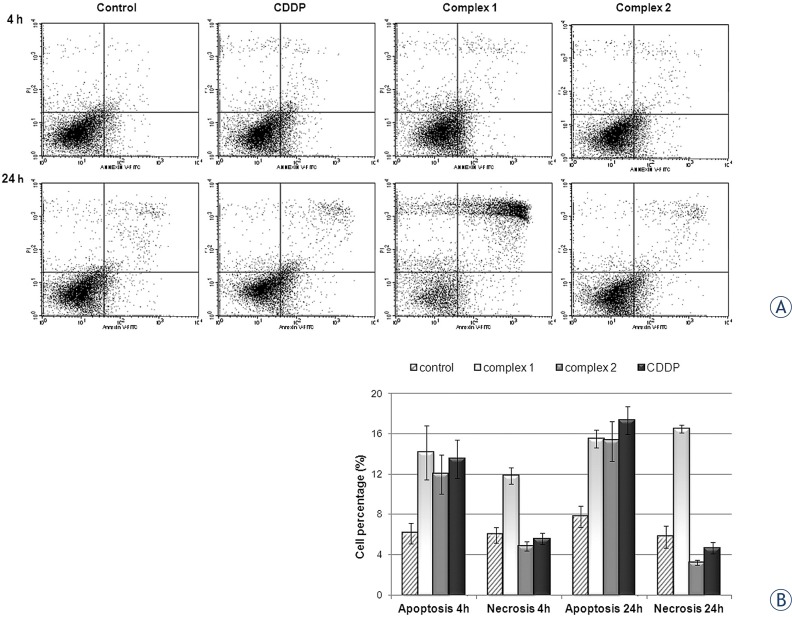
**A** Dot plot diagrams obtained by flow-cytometric analysis of treated HeLa cells after dual staining with Annexin V-FITC and PI. Annexin V-FITC/PI staining was monitored overtime, following 4 and 24 hours in HeLa cells exposed to complex **1**, **2** or CDDP at concentrations corresponding to IC_50_. Representative dot plots of three independent experiments are given, presenting intact cells at lower-left quadrant, FITC(-)/PI(-); early apoptotic cells at lower-right quadrant, FITC(+)/PI(-); late apoptotic or necrotic cells at upper-right quadrant, FITC(+)/PI(+); and necrotic cells at upper-left quadrant, FITC(-)/PI(+). **B** Apoptosis and necrosis were quanitified by FACS after Annexin V-FITC and PI labeling; bar graphs represent mean ± SD in at least three independent experiments.

Potential of the investigated complex to induce apoptosis in HeLa cells was assessed by flow cytometry using Annexin V-FITC and PI dual staining. Dot plots are presented in Figure 4A, while Figure 4B reports the results of a representative experiment as percentages of apoptotic cells (Annexin V-FITC positive and PI negative) and necrotic cells (Annexin V-FITC negative and PI positive) measured periodically at 4 and 24 h. Data obtained indicated that complex **2** caused 15.5% of apoptosis in HeLa cells following 24 h of action, while the percentage of necrotic cells was negligible. Kinetics, as well as the degree of apoptosis induction, was comparable to CDDP. Complex **1** at concentration of IC_50_ initiated early apoptotic cell death after 4 and 24 h action, where apoptotic cell population represented 14.2% and 15.5% of total cells, respectively. Though, after 24 h of action more than 50% of total cell population underwent cell death in either apoptotic or necrotic manner.

### Determination of the platinum(II) binding to intracellular DNA and proteins

**FIGURE 5.**
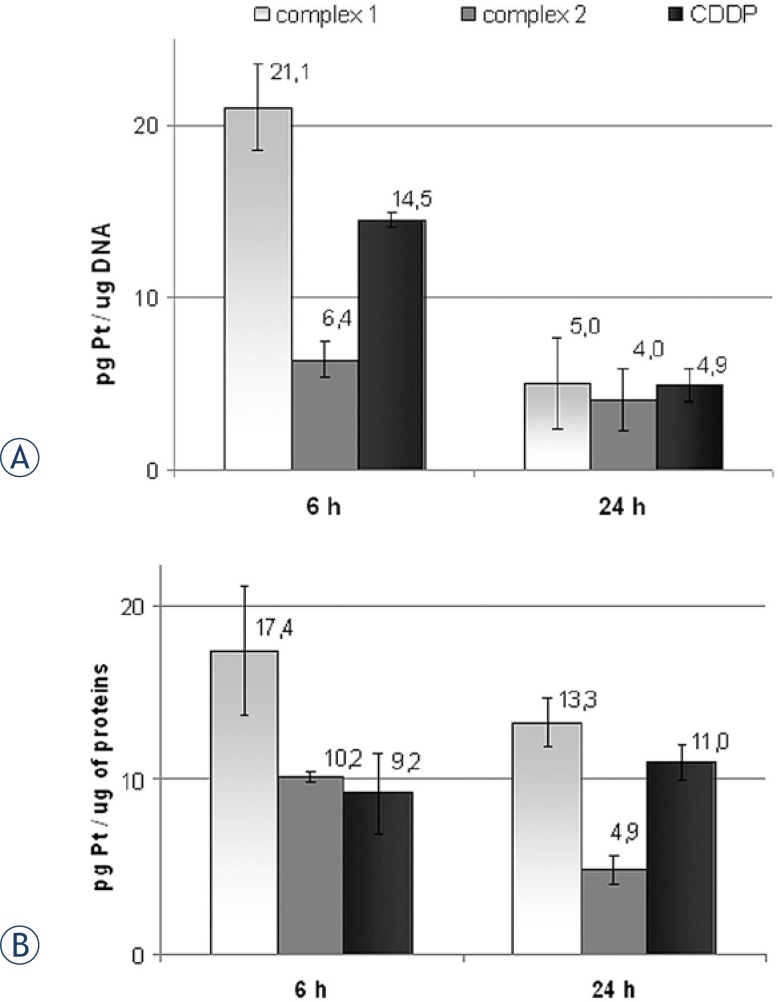
Diagrams presenting quantitative determination of platinum(II) content in DNA and proteins in HeLa cells, obtained by ICP-OES analysis, following 6 h and 24 h of action of **1**, **2** or CDDP; **A** Platinum content in cellular DNA; **B** Platinum content in cellular protein fraction (pg Pt/mg proteins). Bar graph represent mean ± SD of three independent experiments.

Intracellular platinum(II) distribution among DNA and protein fractions in HeLa cells treated with equitoxic concentrations of investigated complexes for 6 and 24 h, was analyzed using ICP-OES analysis, and results are presented in Figure 5. Levels of platinum(II)-DNA binding (Figure 5A), varied between the investigated complexes, especially following short-term (6 h) treatment, when platinum content (pg Pt/mg DNA) decreased in order: 21 ± 2.5 (complex **1)**; 14.5 ±0.4 (CDDP) and 6.4±1.1 (complex **2**). Both CDDP and **1** seemed to be more effcient in promoting cellular DNA binding comparing to complex **2,** though differences in double-stranded DNA platination affinity between complex **2** and CDDP were in accordance to the recent study.^34^ Platinum(II)-DNA content, decreased in time-dependent manner, and reached comparable levels following 24 h of action. Results of the ICPOES analysis of platinum(II) content in the protein fraction (Figure 5B), indicated that **1** exhibited the highest affinity for protein binding following both 6 h and 24 h treatment, while **2** exhibited the lowest binding affinity. Time dependent decrease of protein binding, indicated reversible nature of interactions of **1** and **2**, oppositely to CDDP.

### Protein and mRNA expression of ERCC1

**FIGURE 6.**
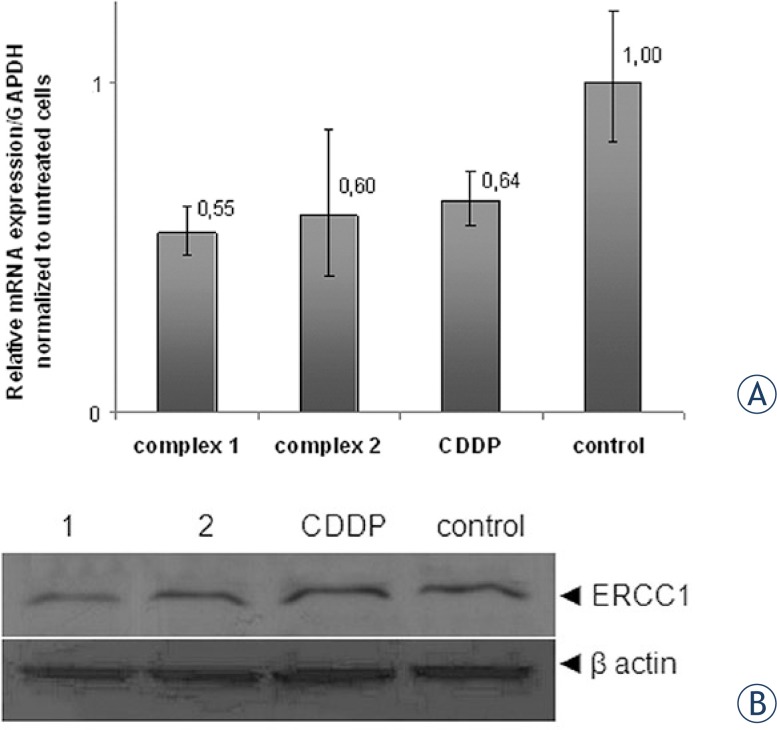
**A** Results of the qRT-PCR analysis of ERCC1 mRNA presented as diagrams showing relative expression level of ERCC1 mRNA, normalized with the GAPDH; Bar graph represent mean ± SD of three independent experiments; **B** Protein expression levels of ERCC1 determined by Western blot, and normalized with b-actin. Tested agents **1**, **2** and CDDP were applied at concentration of 0.5×IC_50_. Western blot results show one representative experiment selected of three.

DNA excision repair protein ERCC1 is an important component of NER (Nucleotide Excision Repair) which is primarily induced in the repair of bulky platinum-DNA adducts.^35^ In order to evaluate whether investigated complexes induce ERCC1-dependent cell response as the result of cytotoxic DNA lesions, we investigated mRNA and protein expression level of ERCC1. Data obtained on HeLa cells after 6 h of continuous treatment with equitoxic concentrations of tested *trans*-platinum complexes or CDDP indicated negative modulation of ERCC1 expression on both mRNA and protein levels (results presented in Figure 6). Complexes **1**, **2** and CDDP decreased ERCC1 mRNA level for 45%, 40% and 36%, respectively, comparing to the non treated control (Figure 6A). Western blot analysis (Figure 6B) showed reduction of ERCC1 protein levels, following both *trans*-complexes **1** and **2** action, while there were no obvious changes associated with CDDP treatment.

### Tube formation assay (*in vitro* angiogenesis assay)

**FIGURE 7.**
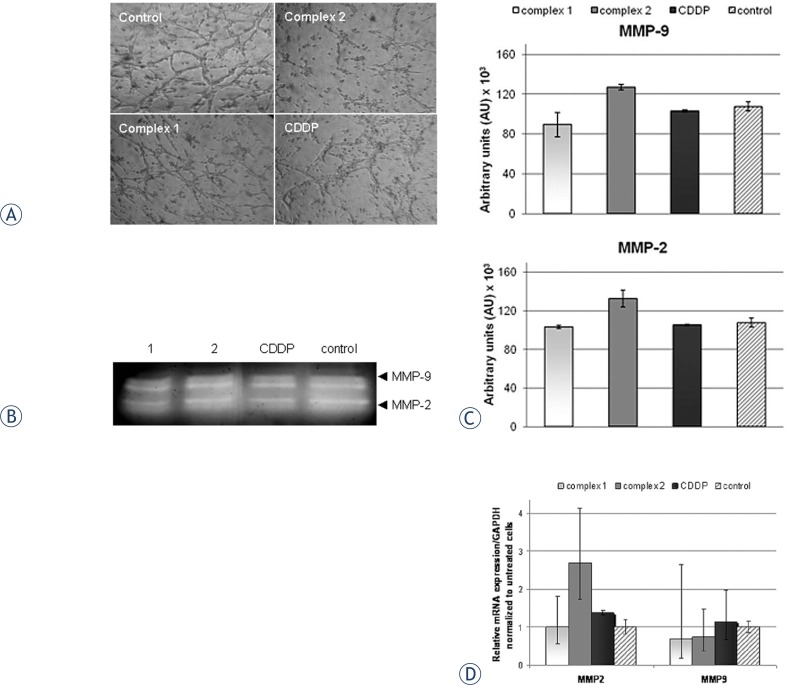
**A** Micrographs of tube formation of MS1 cells, taken following 24 h treatment with sub-toxic concentrations (0.03×IC_50_) of complexes **1**, **2** or CDDP, or without treatment (control cells); **B** Results of the gelatin zymography following 6 h treatment with 0.5×IC_50_ of **1**, **2** or CDDP. Supernatants were collected and equal amounts of proteins were analysed by zymography. Representative of three independent experiments; **C** Diagram presenting quantification of catalytic activity of secreted MMP-2 and MMP-9 in treated HeLa cells, obtained by quantitative analysis of zymograms, using Image J software. Data are expressed in arbitrary units as mean ± standard deviation of three independent experiments; **D** Results of the qRT-PCR analysis of the MMP-2 and MMP-9 mRNA expression level in HeLa cells after 6 h treatment with **1**, **2** or CDDP, at concentration 0.5×IC_50_. Each bar represents the average (±SD) of three independent experiments.

In order to determine the potency of the investigated complexes to restrict the angiogenesis of cancer cells, we performed an *in vitro* tube formation assay in mouse endothelial cells MS1. In our experiment, MS1 endothelial cells were treated with sub-toxic concentrations of the investigated complexes in order to distinguish among growth inhibitory effect and their potential to inhibit the formation of tube-like structures. Antiangiogenic effect was observed for both tested *trans*-platinum complexes, and results are presented in Figure 7A. *Trans*-complexes, particularly complex **2,** showed inhibitory effect on the formation of cell-cell contact and tube-like structures, at very low sub-toxic concentration corresponding to 0.03×IC_50_, while CDDP did not exhibit any significant effect in this assay.

### Gelatin zymography and determination of MMP-9 and MMP-2 expression on mRNA level

We investigated whether tested complexes were able to modulate mRNA expression of MMP-2 and MMP-9 or affect their gelatinolitic activity *in vitro*. The effect of **1** and **2** on the activity of the secreted forms of MMP-2 and MMP-9 in HeLa cells, was examined following 6 h action, by gelatin zymography and the results are presented in Figure 7B. Quantitative analysis of the gelatin zymography was performed by Image J software, and is presented in Figure 7B. Results obtained indicated that complex **1** induced moderate decrease of gelatinolitic activity of MMP-2 and MMP-9 in comparison to the control (non treated cells), when applied at concentration of 0.5×IC_50_, (Figure 7C). CDDP failed to show effect in this assay, while complex **2** induced minor enhancement of metalloproteinases activity. Results obtained by qRT-PCR indicated that t*rans*-complexes reduced level of MMP-9 mRNA, comparing to the control, following 6 h treatment (Figure 7D). Complex **2** and CDDP, when applied at equitoxic concentrations corresponding to 0.5×IC_50_, upregulated MMP-2 mRNA, while complex **1** did not induce obvious alteration of MMP-2 mRNA expression.

## Discussion

In our previous study we have reported synthesis, structural characterization and cytotoxic potential of two *trans*-platinum complexes of structural formula *trans*-[PtCl_2_(n-acetylpyridine)_2_] (n = 3 or 4, complex **1** or **2**, respectively), revealing significant cytotoxic potential of complex **2** on several tumor cell lines, with the highest potential in HeLa cells.^23^ In the current study we investigated the mechanism underlying *in vitro* antitumor activity of both isomers, in order to understand possible relations to their structural differences, such as position of the acetyl substituent on pyridine ligand. Study was performed in comparison to CDDP as referent compound.

Cytotoxicity evaluation in MRC-5 cells, which were used as *in vitro* non-cancerous cell model, showed feature of the tested *trans*-platinum isomers to exert less toxicity in MRC-5, than in HeLa. Particularly complex **2** with 4-acetylpyridine, exhibited significant cytoselective potential toward neoplastic cells (HeLa) relative to normal cells (MRC-5), cells (p < 0.001), comparing to CDDP (p > 0.05).

According to the results of flow cytometry, antiproliferative action of complex **1,** was associated to minor cell cycle arrest in the G0/G1 and S phase, and consequent initiation of cell death. When tested at equitoxic concentrations (IC_50_), complex **1** induced significant percentage of necrotic cells, comparing to complex **2** and CDDP, observed already after 4 h of action. Complex **2** exhibited the rate and kinetics of apoptosis induction similar to that of CDDP.

Mechanism of anticancer activity of platinum drugs is believed to be associated with their binding with cellular DNA. The level of DNA-binding is considered to provide more meaningful information (related to activity) than total cellular uptake especially as platinum drugs may undergo complexation with cellular platinophiles, so that only a very small fraction of the drugs actually binds with DNA.^36^

The results of the present work, demonstrating nuclear DNA binding affinity of compounds **1** and **2**, are consistent with the hypothesis that the minor structural modifications of carrier ligands in *trans*-mononuclear platinum complexes could modulate the DNA binding afinity, resulting in the altered biological (pharmacological) activity of these new platinum complexes in tumor cells, relative to CDDP.^37^ Results of the analysis of the actual platination of DNA, presented in terms of pg Pt/mg DNA, showed that **2** exhibited the lowest DNA binding following 6 h treatment, with platinum content being less by a factor of approximately threefold comparing to **1**, though reaching similar level of DNA binding as **1** and CDDP following 24 h treatment. Structure-activity correlation suggests that the acetyl-group in the para-position on the planar pyridine rings (4-acetylpyridine) in complex **2**, may additionaly hamper positioning of the non-leaving moieties in the adducts of this analogue, that would be entirely favorable for its interaction with the double helix.^37^ It should be noted that complex **2** platinum-DNA level just slightly decreased during 24 h treatment comparing to complex **1** and CDDP, suggesting on the other side sustainable nature of platinum-DNA lesion. Different studies support the assumption that long-lived DNA-adducts, formed by mononuclear *trans*-platinum(II) complexes containing planar ligands such as quinoline or pyridine constitute potential cytotoxic lesions.^38^ The higher cytotoxicity of **2** comparing to **1**, may be attributed to its ability to form different DNA conformational distorsions and lesions which are differentially processed by DNA damage recognition/repair proteins, and to the ability of compound to induce different cellular response.^21^ Lower level of unfavorable interactions with proteins may be additional determinant of enhanced cytotoxicity of complex **2** in comparison to **1**.

On the other side, meta-position of the acetyl substituent on the pyridine ring (3-acetylpyridine) in complex **1,** allowed reactivity with intracellular DNA and proteins, though in reversible manner.

Complex **1** platinum levels in cellular DNA decreased from 6 h to 24 h time points, indicating that **1** treated cells partially recovered from the initial cytotoxic stress, which may be due to an early DNA damage response and removal of the platinum-DNA lesions.^13^

In order to evaluate role of DNA-damage repair in mediating differences in cytotoxicity of the tested complexes, we further evaluated expression of ERCC1 on mRNA and protein level. Nucleotide excision repair is one of the DNA-repair mechanisms primarily activated in response to cisplatin induced genotoxic stress.^39^–^42^ In the case of CDDP, cycle cycle arrest in the phase G0/G1 (also caused by investigated complex **1**) and G2/M arrest, may be indicative for activation of NER repair proteins.^20^ Nevertheless, western blot and gene expression analysis in the current study, revealed reduction of ERCC1 mRNA and protein levels, following short-term treatment with **1** and **2**, while there was discordance in mRNA and protein levels following CDDP treatment. It is likely that ERCC1 might not play fundamental role in mediating sensitivity to *trans*-platinum complexes, as well as to the investigated complexes in the current settings, but additional studies need to be directed toward understanding of the molecular mechanism underlying expression status of ERCC1 (mRNA and protein), and its correlation to *trans*-platinum-based drug sensitivity.^15^,^43^

In the separate part of our study we investigated the potential of tested complexes to modulate processes related to angiogenic and metastatic potential of tumor cells *in vitro*. Pathological angiogenesis is a hallmark of cancer and represents an important step in the development of metastasis.^44^–^46^ Significant efforts in the area of anticancer drug research are focused on the development of a drug, which would be able to limitate angiogenesis of cancer cells. Study in MS1 cells revealed potential of **2** to inhibit formation of tube-like structures and tumor cell-cell contacts, at very low subtoxic concentration (0.03 IC_50_), while CDDP failed to show effect in this assay. Our results indicated potential of complex **2** to act on multiple processes in cancer cells, and that exposure to DNA-damaging agents at subtoxic concentrations may alter tumor cell behavior. More direct experiments would be required to confirm the observed antiangiogenic potential of **2***in vitro*.

Matrix metalloproteinases 2 and 9, which are frequently over expressed in tumor cells, play a critical role in modulation of extra cellular matrix, and its role in tumor cell migration, formation of tumor cell contacts and angiogenesis transition is extensively investigated. Thus, MMPs represent a promising target for antitumor drug design.^25^,^45^ Our investigations of the effect of tested complexes on gelatinolitic activity of secreted matrix metalloproteinases MMP-2 and MMP-9 and their mRNA expression levels, showed that both **1** and **2** caused decrease of MMP-9 mRNA, for 6 h action, though for the time point observed (6 h), inhibitory effect on the enzyme activity level was minor.^44^ Only complex **1** showed moderate inhibitory effect on MMP-2 and MMP-9 activity. Reduction of functional levels of MMP-9 by **1** and **2** was in correlation to the reduction of the enzyme mRNA-level, which might represent the indirect effect of genotoxic stress.

Although two investigated complexes of structural formula *trans*-[PtCl_2_(n-acetylpyridine)_2_] (n = 3 or 4) represent close structural isomers, their interactions with cellular targets and consequently induced cellular responses are different. Results obtained suggest that structural settings play a fundamental role, since they are responsible for the interaction between *trans*-Pt drug and cellular DNA and proteins and the consequent biological effects. Higher cytotoxicity of **2** compared to the **1** analogue, may be attributed to its ability to form different DNA-lesions, produce different cellular effects related to damage-precessing and signal activation pathways, and induce multiple cellular responses. Our study demonstrated that complex **2** is particularly interesting since it exhibited cytotoxic and apoptotic potential in HeLa cells comparable to that of CDDP, though showing differences in terms of reactivity to DNA and proteins, cytoselectivity toward tumor cells and potential for *in vitro* angiogenesis inhibition. Further explorations are needed to determine a possible differential mechanism of action and elucidate antitumor potential of comple*x in vivo.*

Altogether, properties of complexes of structural formula *trans*-[PtCl_2_(n-acetylpyridine)_2_] (n = 3 or 4), encourage further investigation of substituted *trans*-platinum pyridines in search for compound with different modalities of action toward cancer cells in comparison to CDDP.
